# A comparison of conscious sedation and local anesthesia for thrombectomy in acute ischemic stroke: a multicenter study

**DOI:** 10.3389/fneur.2024.1416146

**Published:** 2024-08-01

**Authors:** Aysenur Onalan, Erdem Gurkas, Cetin Kursad Akpinar, Özlem Aykaç, Turkan Acar, Bilgehan Acar, Zehra Uysal Kocabaş, Hasan Doğan, Ferhat Balgetir, Sule Kavak Genc, Ahmet Yabalak, Atilla Ozcan Ozdemir

**Affiliations:** ^1^Stroke Center, Istanbul Kartal Dr. Lutfi Kirdar City Hospital, Istanbul, Türkiye; ^2^Stroke Center, Samsun Training and Research Hospital, Samsun, Türkiye; ^3^Stroke Center, Faculty of Medicine, Eskişehir Osmangazi University, Eskişehir, Türkiye; ^4^Stroke Center, Department of Neurology, Faculty of Medicine, Sakarya University, Sakarya, Türkiye; ^5^Stroke Center, Faculty of Medicine, Firat University, Elazig, Türkiye; ^6^Stroke Center, Faculty of Medicine, Duzce University, Ankara, Türkiye

**Keywords:** conscious sedation, local anesthesia, endovascular treatment, large vessel occlusion, propensity score matching

## Abstract

**Introduction:**

Ischemic cerebrovascular disease (ICVD) is a serious health problem in which brain tissue suffers from hypoxic damage due to obstruction in cerebral vessels. Mechanical thrombectomy is a commonly used method in the treatment of these patients. However, the effects of local anesthesia (LA) and conscious sedation (CS) during thrombectomy are still unclear. We evaluated whether there was a relationship between the two anesthesia regimens in terms of 90-day modified Rankin Scale (mRS) scores.

**Methods:**

In this study, a retrospective observational study was conducted to evaluate the effects of LA and CS used during mechanical thrombectomy in four comprehensive stroke centers among ICVD patients. Patients were divided into the LA group and the CS group. Statistical analysis was performed before and after 1:1 matching under propensity score matching (PSM) analysis. The primary outcome measure was an mRS score of 0–2 at 90 days. Secondary outcomes were procedure times, recanalization rates, symptomatic and asymptomatic hemorrhage rates, and procedural complications.

**Results:**

A total of 193 patients (118 patients with LA and 75 patients with CS) were included in the final analysis before PSM. After 1:1 PSM, 98 patients—49 patients from each group—were included in the study. There was no difference in clinical outcomes between the LA- and CS-applied groups (*p* = 0.543). When blood pressure (BP) values at follow-up after endovascular treatment (EVT) were compared, the lowest systolic and lowest diastolic BP were found to be lower in the CS group (*p* = 0.001 and *p* = 0.009). There was no significant difference between the two groups in terms of recanalization rates, symptomatic intracranial hemorrhage (sICH) rates, 90-day mRS, and procedure-related complication rates (*p* = 0.617, *p* = 0.274, *p* = 0.543, and *p* = 1.000).

**Conclusion:**

This study did not reveal the superiority of CS applied during EVT on 90-day mRS, sICH, recanalization rates, or procedural complications. However, the risk of developing hypotension during the CS application was found to be high.

## Introduction

Mechanical thrombectomy is the most appropriate treatment option for acute ischemic stroke (AIS) patients with large vessel occlusion (1). This interventional method aims to recanalize occluded cerebral vessels and restore blood flow to the ischemic area (2). Thrombectomy is especially important for patients who do not respond to conventional intravenous thrombolytic therapy or who are not amenable to thrombolytic therapy. However, patients’ comfort and cooperation during the thrombectomy procedure may affect the success of the treatment. Thrombectomy is performed using one of the three anesthesia methods: local anesthesia (LA), conscious sedation (CS), or general anesthesia (GA). There is no definitive conclusion about the optimal anesthesia regimen (3). The debate continues regarding the effects of these three approaches on patients’ level of cooperation, level of anxiety during the procedure, and outcomes. GA is not the preferred anesthesia method of operators in the real world because it is time-consuming (4). LA aims to ensure patient awareness, cooperation during the procedure, and minimal pain at the procedure site during the procedure. On the other hand, CS helps patients remain more sedentary and reduces the level of anxiety during the procedure (5). Therefore, it is important to better understand the effects of LA and CS on the mechanical thrombectomy procedure in AIS patients and to elucidate the advantages and disadvantages of these two approaches.

To reduce selection bias, we compared the outcomes of all consecutive patients with anterior circulation large vessel occlusion who underwent EVT at four centers: two comprehensive stroke centers that consistently applied the LA strategy and two comprehensive stroke centers that consistently applied the CS strategy. The primary outcome of this study was whether there was a difference in LA and CS in terms of 90-day modified Rankin Scale (mRS) scores between the two groups during the thrombectomy procedure. The secondary outcomes included recanalization rates, symptomatic and asymptomatic hemorrhage rates, procedure times, and procedure complication rates.

## Methods

### Patient population

A total of 333 patients who underwent mechanical thrombectomy with LA and CS performed at four comprehensive stroke centers between 1 March 2021 and 1 January 2022, were consecutively collected and retrospectively analyzed. During this period, two centers performed mechanical thrombectomy under LA and two centers performed mechanical thrombectomy under CS.

This study included patients with cerebral infarction caused by acute occlusion of large arteries of the anterior system (ICA, MCA M1-M2 segment) who were admitted to the emergency department within 6 h after stroke onset. It also included patients presenting between 6 and 16 h after last seen well who met the DAWN criteria. Other inclusion criteria include patients who underwent mechanical thrombectomy with a pre-stroke mRS of 0–1, were over the age of 18, and underwent either LA or CS. The study excluded patients with posterior system (vertebrobasilar system) infarction, previous mRS ≥ 2, and missing data, or patients receiving general anesthesia during mechanical thrombectomy. This study was approved by the local Ethics Committee of Health Sciences University, Kartal Dr. Lutfi Kırdar City Hospital (Decision no: 2023/5l4/254/28, Date: 19 July 2023). Informed consent forms were obtained from the patients or legal representatives of the patients for participating in the study, which was conducted in accordance with the Declaration of Helsinki.

A total of 128 of the 333 patients did not meet the inclusion criteria. The remaining patients were divided into two groups according to the anesthesia applied: LA and CS patients. In the group receiving LA, 10 mL of 2% prilocaine was applied to the peripunctural site just before the procedure, and no sedative agent was used. The group receiving CS received dexmedetomidine at a rate of 0.2–0.5 mcg/kg/h, administered just before the procedure.

### Outcomes

The primary outcome variable was to evaluate whether there was a difference in mRS 0–2 at 90 days between these two groups. Secondary outcome variables included procedure times, recanalization rates, symptomatic and asymptomatic hemorrhage rates, and procedural complications. Symptomatic intracerebral hemorrhage was defined according to the ECASS III criteria (6).

Clinical status assessment at 90 days was performed face-to-face or by phone call. Clinical status assessors were independent and not involved in this study and were blinded to other clinical details or anesthesia methods.

### Statistical analysis

To evaluate the findings obtained from the study, the data were recorded and analyzed in the statistical package program 25.0. Descriptive statistics are presented as frequency (*n*) and percentage (%) for categorical variables and as median and minimum value (Min)–maximum value (Max) for continuous variables. The chi-square test or Fisher’s exact test was used to compare categorical variables. The compliance of continuous variables with the normal distribution assumption was evaluated with the Kolmogorov–Smirnov test, and since the data were not normally distributed, the comparison of continuous variables between two independent groups was evaluated using the Mann–Whitney U-test. A propensity score matching (PSM) analysis was performed to reduce selection bias inherent in observational studies and limit confounding bias. Individual propensity scores were calculated via a multivariable logistic regression model that took into account clinically relevant predictors of clinical outcome and all variables significantly associated with anesthesia strategy (LA vs. CS). After excluding subjects with a propensity score > 0.95 or < 0.05, patients receiving LA or CS were matched 1:1, with a matching tolerance of 0.02. The propensity score used to compare the two groups was calculated by adjusting for all significant variables in the univariate analysis as well as additional variables likely to influence clinical outcomes. Criteria used for adjustment were as follows: age, admission National Institutes of Health Stroke Scale (NIHSS) and Alberta Stroke Program Early CT Score (ASPECTS) scores, time from symptom onset to presentation, previous intravenous thrombolysis, and occlusion site (right and left). The Wilcoxon rank-sum test was used to evaluate continuous variables before and after EVT. A *p*-value of <0.05 was considered statistically significant.

## Results

**Figure 1:**
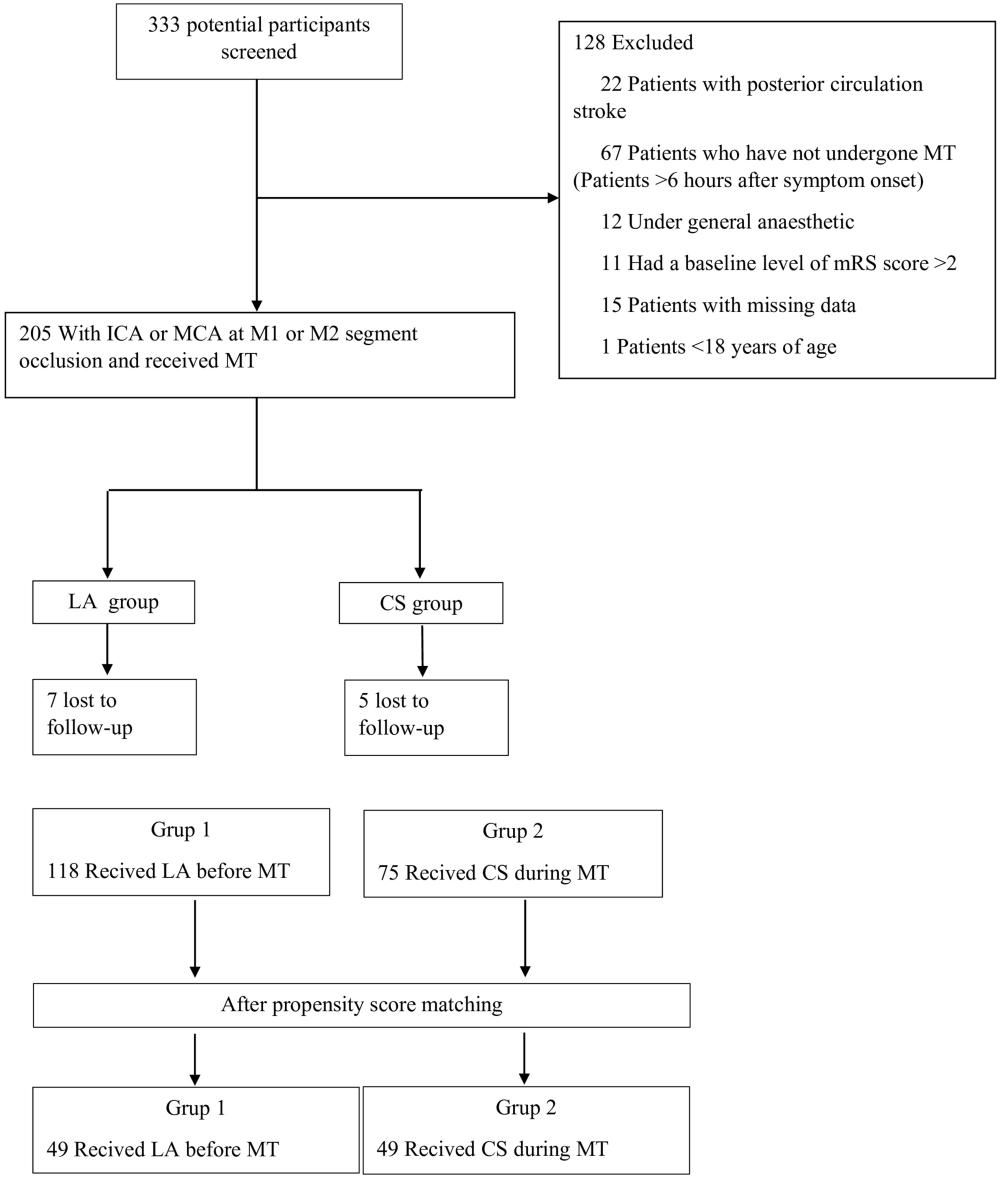
Diagram flowchart of study patients. ICA, internal carotid artery; MCA, middle cerebral artery; mRS, modified Rankin Scale; NIHSS, National Institutes of Health Stroke Scale; CT, computed tomography; MT, mechanical thrombectomy; TT, thrombolytic therapy; Sİ, stent implantation; BDT, balloon dilation therapy; CS, conscious sedation; LA, local anesthesia.

As a result of the review of prospectively maintained databases, 333 patients with anterior circulation stroke were identified. A total of 22 patients were excluded due to a posterior system infarction, 67 patients were excluded because they were admitted after 6 h of stroke onset and did not fulfill the inclusion criteria of the DAWN trial (7), 12 patients received GA, 11 patients had a baseline mRS score > 2, 1 patient was under the age of 18, and 15 patients were excluded because the data were not fully accessible. LA was applied to 125 patients, and CS was applied to 80 patients (Figure 1). Seven patients in the LA group and 5 patients in the CS group were excluded because they were lost to follow-up after 90 days.

As a result, a total of 193 patients (118 patients with LA, 75 patients with CS, and 23 patients fulfilling the inclusion criteria for the DAWN trial) were included in the final analysis before PSM. The median age was significantly older in the LA group. Although the presence of comorbidities was similar between the groups, hyperlipidemia was significantly higher in the LA group (*p* < 0.001). Admission ASPECTS score was significantly higher in the LA group (*p* < 0.001). The time between symptom onset and recanalization and between imaging and groin puncture was significantly longer in the neurosedation group (*p* < 0.001). Admission systolicvBP, peak systolic BP during the procedure, and mean arterial BP were significantly higher in the LA group (*p* < 0.001) (Table 1). In the LA group, one patient switched to neurosedation, and in the CS group, three patients switched to general anesthesia.

**Table 1:** Comparison of baseline, procedure, and outcome characteristics between the two groups before propensity score matching.

	LA (*n* = 118)	CS (*n* = 75)	*p*
Age			
Median (min–max)	72 (28–91)	68 (23–87)	0.006***
Gender (female) *n(%)*	61 (51.7)	29 (38.7)	0.077*
Comorbidity *n(%)*	103 (87.3)	62 (82.7)	0.374*
HT *n(%)*	82 (69.5)	42 (56.0)	0.057*
DM *n(%)*	44 (37.3)	23 (30.7)	0.346*
HL *n(%)*	18 (15.3)	0 (0.0)	<0.001*
CAD *n(%)*	31 (26.3)	20 (26.7)	0.952*
AF *n(%)*	32 (27.1)	25 (33.3)	0.356*
CHF *n(%)*	7 (5.9)	6 (8.0)	0.576*
Stroke history *n(%)*	14 (11.9)	16 (21.3)	0.077*
Antithrombotic therapy *n(%)*			0.105*
Antiaggregant	40 (33.9)	33 (44.0)	
Anticoagulant	23 (19.5)	6 (8.0)	
Both of them	4 (3.4)	1 (1.3)	
Admission NIHSS score, median (min–max)	15 (5–24)	14 (3–26)	0.719***
24th h NIHSS score, median (min–max)	8 (0–24)	10 (0–31)	0.267***
Intravenous Thrombolysis *n(%)*	24 (20.3)	27 (36.0)	0.366*
Admission ASPECTS, median (min–max)	9 (6–10)	8 (3–10)	<0.001***
Occlusion site *n(%)*			0.233*
Right	67 (56.8)	36 (48.0)	
Left	51 (43.2)	39 (52.0)	
Occlusion side *n(%)*			0.024*
MCA M1	79 (66.9)_a_	38 (50.7)_b_	
MCA M2	7 (5.9)_a_	7 (9.3)_a_	
Distal ICA (I, L, T) Cervical_ICA	12 (10.2)_a_	19 (25.3)_b_	
Tandem	20 (16.9)_a_	11 (14.7)_a_	
Onset to admission, median (min–max)	173 (10–780)	124 (6–649)	0.071***
Admission to imaging, median (min–max)	16 (0–62)	15 (4–104)	0.226***
İmaging to puncture time, median (min–max)	36 (10–146)	67 (13–171)	<0.001***
Puncture to recanalization, median (min–max)	43 (15–201)	40 (14–93)	0.330***
Onset to recanalization, median (min–max)	111 (45–243)	137 (58–458)	<0.001***
Thrombectomy technics			0.003*
Aspiration	24 (20.3)_a_	28 (37.3)_b_	
Stent retriever	1 (0.8)_a_	4 (5.3)_a_	
Both of them	93 (78.8)_a_	43 (57.3)_b_	
Admission SBP, mmHg, mean ± SD	167 (110–220)	156 (90–218)	<0.001***
Admission DBP, mmHg, mean ± SD	90 (58–181)	92 (53–129)	0.946***
Onset procedure SBP, mmHg	165 (97–225)	149 (90–200)	<0.001***
Onset procedure DBP, mmHg	90 (50–168)	90 (53–129)	0.735***
During procedure SBP, max, mmHg	175 (110–225)	160 (107–215)	<0.001***
During procedure SBP, min, mmHg	150 (75–180)	123 (71–169)	<0.001***
During procedure DBP, max, mmHg	95 (67–170)	95 (63–168)	<0.001***
During procedure DBP, min, mmHg	80 (50–105)	70 (34–102)	<0.001***
MAP, mean ± SD	110 (66–141)	101 (65–129)	<0.001***
SPO2(%), min	95 (80–110)	93 (78–110)	0.006***
SPO2(%), max	98 (75–105)	98 (80–100)	0.299***

*Pearson chi-square, **Fisher’s exact test, ***Mann–Whitney U-test.

Each subscript letter denotes a subset of anesthesia procedure categories whose column proportions do not differ significantly from each other at the 0.05 level.

LA, local anesthesia; CS, conscious sedation; HT, hypertension; DM, diabetes mellitus; HL, hyperlipidemia; CAD, coronary artery disease; AF, atrial fibrillation; CHF, congestive heart failure; NIHSS, National Institutes of Health Stroke Scale; MCA, middle cerebral artery; ICA, internal carotid artery; IQR, interquartile range; SBP, systolic blood pressure; DBP, diastolic blood pressure; MAP, mean arterial pressure; SPO2, saturation of peripheral oxygen.Bold values are statistically significant.

## Propensity score matching and score-adjusted analysis

Using PSM, 49 patients from each center were matched 1:1 based on baseline characteristics. After matching, all covariates were statistically similar between the two groups except Admission ASPECTS (Table 2). Although the time from symptom onset to groin puncture was similar between the two groups, the time between imaging and groin puncture was significantly longer in the neurosedation group (*p* = 0.001) (Table 2).

**Table 2:** Comparison of baseline, procedure, and outcome characteristics between the two groups after propensity score matching.

	LA (*n* = 49)	CS (*n* = 49)	*p*
Age, median (min–max)	69 (28–91)	69 (31–87)	0.688***
Gender (female) *n(%)*	31 (63.3)	21 (42.9)	0.043*
Comorbidity *n(%)*	46 (93.9)	43 (87.8)	0.487**
HT *n(%)*	36 (73.5)	32 (65.3)	0.381*
DM *n(%)*	18 (36.7)	17 (34.7)	0.833*
HL *n(%)*	3 (6.1)	0 (0.0)	0.242**
CAD *n(%)*	11 (22.4)	14 (28.6)	0.487*
AF *n(%)*	9 (18.4)	18 (36.7)	0.042*
CHF *n(%)*	1 (2.0)	6 (12.2)	0.111*
Stroke history *n(%)*	4 (8.2)	8 (16.3)	0.218*
Antithrombotic therapy *n(%)*			0.573*
Antiaggregant	17 (34.7)	22 (44.9)	
Anticoagulant	7 (14.3)	5 (10.2)	
Both of them	3 (6.1)	1 (2.0)	
Admission NIHSS score, median (min–max)	14 (5–20)	15 (3–26)	0.142***
24th h NIHSS score, median (min–max)	7 (0–20)	9 (0–31)	0.047***
Intravenous Thrombolysis *n(%)*	6 (12.2)	9 (18.4)	0.400*
Admission ASPECTS, median (min–max)	9 (6–10)	9 (6–10)	0.042***
Occlusion site *n(%)*			<0.001*
Right	48 (98.0)	28 (57.1)	
Left	1 (2.0)	21 (42.9)	
Occlusion side *n(%)*			0.461*
MCA M1	34 (69.4)	28 (57.1)	
MCA M2	2 (4.1)	2 (4.1)	
Distal ICA (I, L, T) Cervical_ICA	6 (12.2)	12 (24.5)	
Tandem	7 (14.3)	7 (14.3)	
Onset to admission, median (min–max)	135 (10–505)	135 (6–649)	0.887***
Admission to imaging, median (min–max)	15 (0–62)	17 (5–90)	0.935***
İmaging to puncture time, median (min–max)	49 (10–146)	66 (15–149)	0.001***
Puncture to recanalization, median (min–max)	45 (15–201)	40 (14–93)	0.390***
Onset to recanalization, median (min–max)	121 (45–236)	128 (58–458)	0.092***
Thrombectomy techniques			0.579***
Aspiration	16 (32.7)	17 (34.7)	
Stent retriever	0 (0.0)	1 (2.0)	
Both of them	33 (67.3)	31 (63.3)	
Admission SBP, mmHg, mean ± SD	163 (110–220)	160 (102–218)	0.206***
Admission_DBP, mmHg, mean ± SD	90 (58–139)	92 (55–125)	0.444***
Onset procedure SBP, mmHg	162 (100–225)	160 (102–200)	0.061***
Onset procedure DBP, mmHg	90 (57–168)	90 (60–125)	0.834***
During procedure SBP, max, mmHg	175 (110–225)	165 (110–215)	0.086***
During procedure SBP, min, mmHg	145 (88–180)	130 (71–169)	0.001***
During procedure DBP, max, mmHg	98 (74–170)	98 (67–168)	0.545***
During procedure DBP, min, mmHg	80 (58–104)	71 (47–100)	0.026***
MAP, mean ± SD	108 (72–133)	105 (65–129)	0.145***
SPO2(%), min	95 (84–105)	93 (78–110)	0.009***
SPO2(%), max	99 (77–100)	98 (80–100)	0.093***

*Pearson chi-square, **Fisher’s exact test, ***Mann–Whitney U-test.

LA, local anesthesia; CS, conscious sedation; HT, hypertension; DM, diabetes mellitus; HL, hyperlipidemia; CAD, coronary artery disease; AF, atrial fibrillation; CHF, congestive heart failure; NIHSS, National Institutes of Health Stroke Scale; MCA, middle cerebral artery; ICA, internal carotid artery; IQR, interquartile range; SBP, systolic blood pressure; DBP, diastolic blood pressure; MAP, mean arterial pressure; SPO2, saturation of peripheral oxygen.

Bold values are statistically significant.

**Figure 2:**
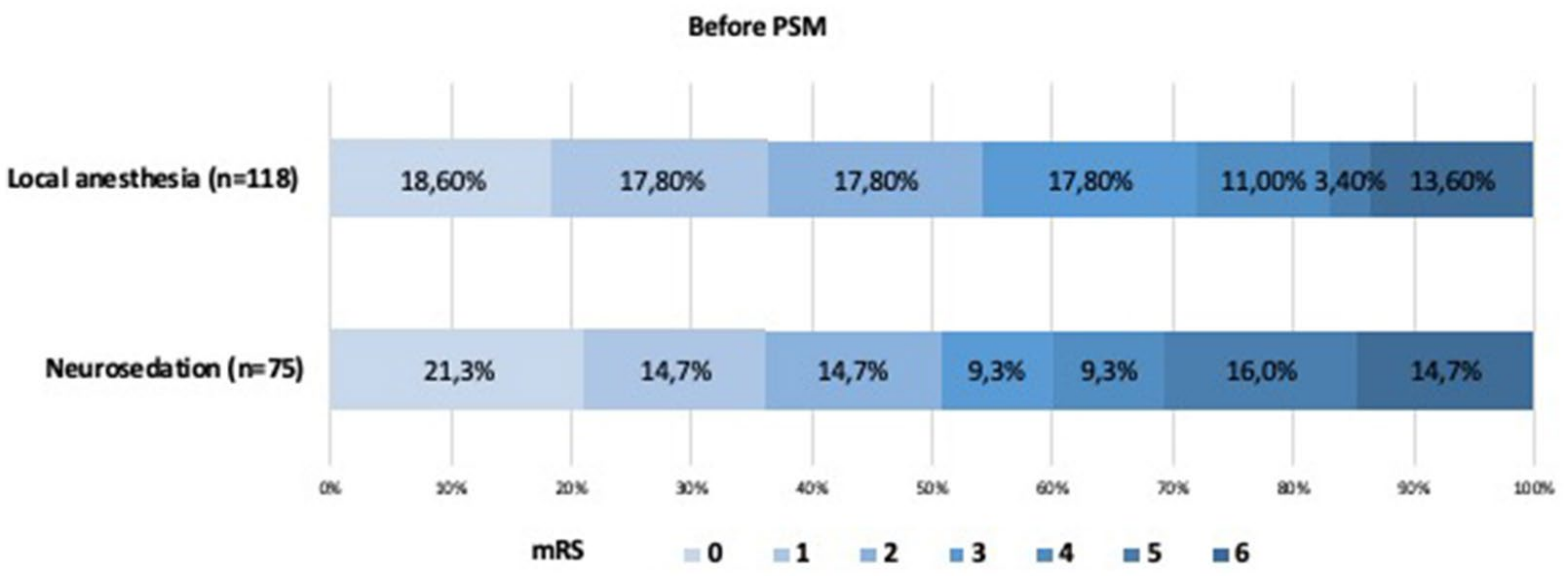
Shift on the 90-day mRS score stratified by LA and CA before propensity score matching.

**Figure 3:**
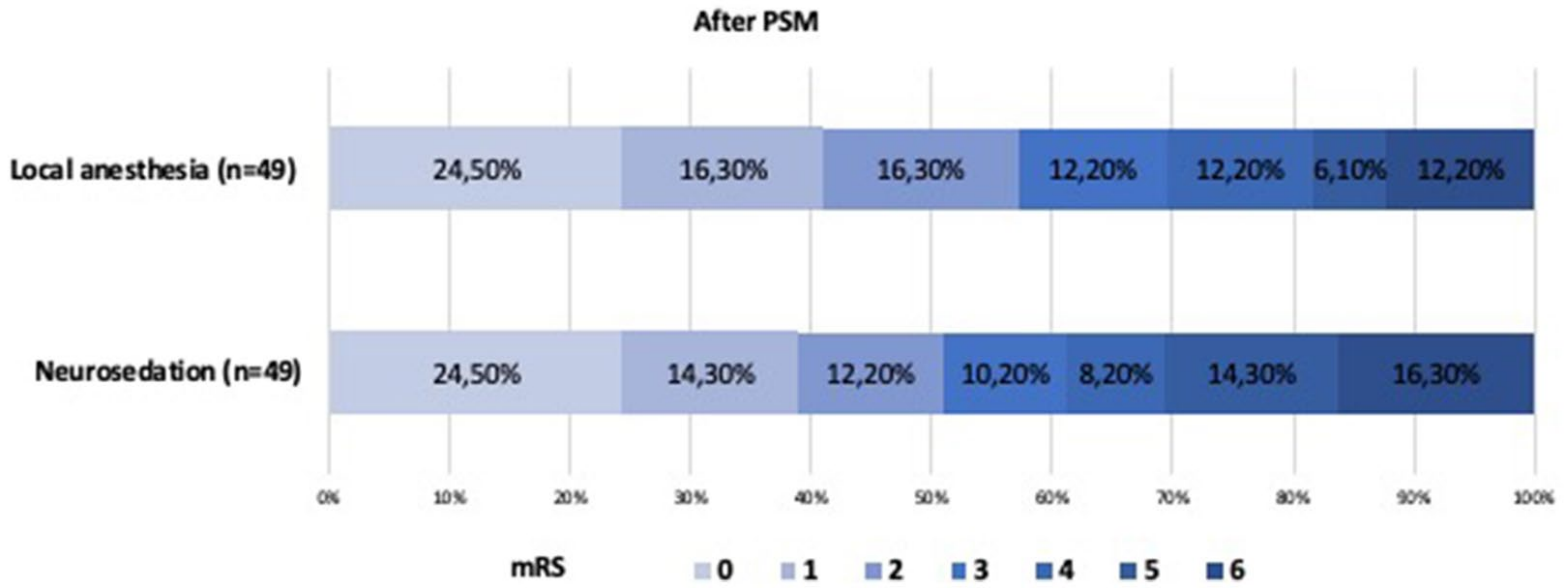
Shift on the 90-day mRS score stratified by LA and CA after propensity score matching.

There were no significant differences in recanalization rates, intracranial hemorrhage (ICH) rates, good outcome (90-day mRS score ≤ 2) rates, or procedure-related complication rates between the two groups (Table 3; Figures 2, 3).

**Table 3:** Group of anesthesia method and outcomes after propensity score matching.

	Local anesthesia (*n* = 49)	Conscious sedation (*n* = 49)	*p*
Symptomatic intracranial hemorrhage, *n* (%)	6 (12.2)	10 (20.4)	0.274
TICI 2b-3, *n* (%)	48 (98.0)	46 (93.9)	0.617
mRS score ≤ 2	28 (57.1)	25 (51.0)	0.543
Procedure-related complications, *n* (%)	0 (0.0)	1 (2.0)	1.000**

*Pearson chi-square, **Fisher’s exact test.

Individual propensity scores were calculated taking into account baseline mRS score, time from symptom onset to entry into the angiography room, admission NIHSS and ASPECTS scores, previous IV thrombolysis, and site of occlusion on angiography. ASPECTS, Alberta Stroke Program Early CT Score; ICA, internal carotid artery; ICH, intracranial hemorrhage; IQR, interquartile range; IV, intravenous; MCA, middle cerebral artery; mRS, modified Rankin Scale; mTICI, modified Thrombolysis in Cerebral Ischemia; NIHSS, National Institutes of Health Stroke Scale; OR, odds ratio.

## Discussion

In this study, the relationship between the anesthesia method and the clinical outcomes of patients who underwent EVT using two different anesthesia strategies in four comprehensive stroke centers was evaluated. The propensity score used to compare the two groups was calculated by adjusting for all significant variables in the univariate analysis as well as additional variables likely to influence clinical outcomes. As a result of the evaluation made after the propensity score analysis, the study shows that LA or CS application does not result in different clinical outcomes in patients with anterior system large vessel occlusion who underwent EVT.

Contradictory results have emerged in studies evaluating the effect of anesthesia management on clinical outcomes in patients who underwent mechanical thrombectomy for AIS under LA or CS as anesthesia management. In two of the studies, LA and CS procedures were associated with worse clinical outcomes (8–11). It is unclear why these conflicting results emerged in these studies. One of the reasons for this may be the use of different generations of thrombectomy devices. Another reason may be the absence of an established protocol for managing blood pressure, oxygen saturation, and carbon dioxide levels during the procedure. A third reason is that the anesthesia technique to be applied to the patients analyzed in these studies was left to the choice of the interventional neuroradiologists’ discretion. In our study, data from centers using different anesthesia techniques were evaluated. This eliminates selection bias in anesthesia selection. Therefore, we believe that our results should be more representative of current clinical practice.

Normally, the time to start endovascular treatment in GA applications is longer than in LA or CS due to the availability of the anesthesia team, long operating room conditions, and the procedure (12, 13). Like LA, CS does not require additional time since it can be performed in the angiography unit and does not require the presence of an anesthesiologist. However, in our study, the time to symptom onset and groin puncture was significantly higher in CS compared to LA. We believe that the reason for this situation is due to in-hospital protocols. We also believe that this situation better reflects real-life data compared to more standard randomized studies. This finding should be used as a precursor to improving in-hospital protocols to reduce treatment delays.

In patients with AIS, a 10-mmHg increase in systolic blood pressure above 150 mmHg increased the death rate by 3.6%, whereas a 10-mmHg decrease increased the death risk by 17.9% (14). Studies have shown that there is a strong relationship between blood pressure values and good clinical outcomes in patients receiving endovascular treatment (3). In a retrospective analysis of patients who underwent thrombectomy with CS, a ≥ 10% decrease from baseline in mean arterial pressure (MAP) was found to be the highest risk factor for poor outcomes. In addition, every 10 mmHg decrease in MAP <100 mmHg before reperfusion was associated with a worse prognosis (15). However, in studies comparing LA and CS, it was not evaluated whether there were blood pressure changes (8–11). To date, there are no studies describing periprocedural BP changes in patients treated under LA. In our study, the effect of LA and CS on arterial blood pressure was also evaluated. There was no significant difference when the admission systolic and diastolic blood pressures were compared between the two groups. When blood pressure values at follow-up after endovascular treatment were compared, the lowest systolic and lowest diastolic blood pressures were found to be lower in the CS group. It should be kept in mind that hypotension may not develop only in procedures where GA is applied, but also in patients who undergo CS.

During endovascular treatment, it is important to avoid both hypertension and hypotension. Hypertension is associated with adverse clinical outcomes, including mortality and symptomatic intracranial hemorrhage (sICH) (16). There was no significant difference between the two groups in our study in terms of recanalization rates, ICH rates, 90-day mRS, and procedure-related complication rates.

Our study also has some limitations. First, patients were not randomized according to the anesthesia type. Although our data were collected prospectively, retrospective analysis was performed. Second, we did not use data on the patients’ collateral circulation. Third, only patients who underwent LA and CS were evaluated in this study. Fourth, we used propensity score analysis to minimize variation in baseline characteristics, which resulted in our sample size being relatively small and, in turn, limited the study’s ability to detect differences between groups. Before PSM, there was no difference in the occlusion site. After PSM, almost all (98%) of the patients with LA had right-sided LVO. Although it is possible that patients without aphasia were mostly included in the LA group, this may cause a bias. It should be noted that patients with right hemisphere involvement do not have aphasia; instead, they are predominantly associated with the development of mania, depression, psychosis, hallucinations, personality changes, anxiety, and dissociative states (17). As a result, right hemisphere involvement may also have an impact on the discomfort of operators during procedures, similar to patients with aphasia.

## Conclusion

In this study, we observed that there was no difference in terms of good clinical outcomes in endovascular thrombectomy patients who underwent both LA and CS. The results of the study may guide clinicians in the selection of anesthesia methods used during mechanical thrombectomy in the treatment of patients with AIS. Additionally, the results of this study will encourage us to expand the sample size in future studies. Current findings need to be confirmed by further prospective studies.

## Data availability statement

The original contributions presented in the study are included in the article/Supplementary material, further inquiries can be directed to the corresponding author.

## Ethics statement

The studies involving humans were approved by Kartal Dr. Lutfi Kırdar City Hospital Clinical Research Ethics Committee (Decision no: 2023/5l4/254/28, Date: 19 July 2023). The studies were conducted in accordance with the local legislation and institutional requirements. Written informed consent for participation was not required from the participants or the participants’ legal guardians/next of kin in accordance with the national legislation and institutional requirements.

## Author contributions

AO: Data curation, Formal analysis, Funding acquisition, Methodology, Project administration, Resources, Software, Validation, Visualization, Writing – original draft, Writing – review & editing. EG: Conceptualization, Funding acquisition, Methodology, Validation, Writing – review & editing. CA: Funding acquisition, Investigation, Methodology, Validation, Writing – review & editing. ÖA: Data curation, Investigation, Methodology, Writing – review & editing. TA: Data curation, Resources, Validation, Writing – review & editing. BA: Supervision, Visualization, Writing – review & editing. ZK: Data curation, Investigation, Project administration, Writing – review & editing. HD: Data curation, Software, Supervision, Writing – review & editing. FB: Resources, Software, Writing – original draft. SG: Methodology, Visualization, Writing – review & editing. AY: Data curation, Validation, Writing – review & editing. AOO: Project administration, Supervision, Writing – review & editing.
